# Train-Time and Test-Time Computation in Large Language Models for Error Detection and Correction in Electronic Medical Records: A Retrospective Study

**DOI:** 10.3390/diagnostics15141829

**Published:** 2025-07-21

**Authors:** Qiong Cai, Lanting Yang, Jiangping Xiao, Jiale Ma, Molei Liu, Xilong Pan

**Affiliations:** 1 Department of Social Medicine and Health Education, School of Public Health, Peking University, Beijing 100191, China; xqiongc@stu.pku.edu.cn (Q.C.);; 2 Department of Biostatistics, Peking University Health Science Center, Beijing 100191, China

**Keywords:** medical record quality, large language models, electronic medical records, quality control, train-time, test-time, assembly model

## Abstract

**Background/Objectives:** This study examines the effectiveness of train-time computation, test-time computation, and their combination on the performance of large language modeling applied to an electronic medical record quality management system. It identifies the most effective combination of models to enhance clinical documentation performance and efficiency. **Methods:** A total of 597 clinical medical records were selected from the MEDEC-MS dataset, 10 of which were used for prompt engineering to guide model training. Eight large language models were employed for training, focusing on train-time computation and test-time computation. Model performance on specific error types was assessed using precision, recall, F1 score, and error correction accuracy. The dataset was divided into training and testing sets in a 7:3 ratio. The assembly model was created using binary logistic regression for assembly analysis of the top-performing models. Its performance was evaluated using area under the curve values and model weights. **Results:** GPT-4 and Deepseek R1 demonstrated higher overall accuracy in detecting errors. Models that focus on train-time computation exhibited shorter reasoning times and stricter error detection, while models emphasizing test-time computation achieved higher error correction accuracy. The GPT-4 model was particularly effective in addressing issues related to causal organisms, management, and pharmacotherapy, whereas models focusing on test-time computation performed better in tasks involving diagnosis and treatment. The assembly model, focusing on both train-time computation and test-time computation, outperformed any single large language model (Assembly model accuracy: 0.690 vs. GPT-4 accuracy: 0.477). **Conclusions:** Models focusing on train-time computation demonstrated greater efficiency in processing speed, while models focusing on test-time computation showed higher accuracy and interpretability in identifying and detecting quality issues in electronic medical records. Assembling the train-time and test-time computation strategies may strike a balance between high accuracy and model efficiency, thereby enhancing the development of electronic medical records and improving medical care.

## 1. Introduction

Electronic medical records (EMRs) are multimedia documents used to electronically record necessary information about a patient’s condition, but the quality of EMRs is still a major concern [1]. During clinical documentation, EMRs are often affected by issues such as record duplication, information errors, and inconsistencies [2,3]. Medical quality serves as the core competitiveness of hospitals, and the quality of medical records reflects both the quality of medical care and the management level of the hospital. Studies have shown that improving the quality of EMRs not only increases the adoption of EMRs in hospitals worldwide but also greatly improves the quality of medical care in hospitals and helps reduce doctor–patient conflicts [3].

Large language models (LLMs) have shown promising potential in improving the quality of EMRs [4,5]. Currently, some studies have focused on detecting and correcting medical record errors using traditional natural language processing techniques [6]. In most cases, clinical diagnostic reasoning is considered to require rich medical expertise and strong logical reasoning skills [7,8]. However, methods based on rule matching or traditional machine learning models tend to perform poorly in logical reasoning and contextualized reasoning. LLMs, with powerful abilities in semantic understanding, knowledge representation, and logical reasoning, have advantages in error detection and correction in EMRs [9,10]. Recent studies have shown that the application of LLMs has achieved better results in the processing of medical texts [11]. With the evolution of technology, LLMs show different focuses on computational resources demand during train-time computation and test-time computation [12]. Train-time computation is the process in which a model acquires knowledge and capabilities by learning a large amount of data and intense computation during the training phase. When the model training is completed and the parameters are fixed, it is suitable for task clarity, massive data, and anomaly recognition [13]. Meanwhile, test-time computation is the process in which the model improves its performance of the model in the inference phase by chaining inference through interaction with the user or specific instructions. The model focuses on optimizing the model structure at each inference, and then iterates itself to put more resources into the inference process, which is suitable for complex analysis of multi-source data and clinical decision-making [14,15]. One study has demonstrated that prompt-based integration of train-time computation and test-time computation in LLMs can break through the limitation of model inference performance [16,17]. Although the synergistic distribution of computational resources is beneficial to enhancing the performance of LLMs, the impact of computation allocation strategies on EMR error identification and correction remains unclear.

To compare the capabilities of LLMs in detecting and correcting errors in EMRs, we evaluated two categories of LLMs with different computational resource allocation: models focusing on train-time computation and models focusing on test-time computation. We selected the generic LLMs for this comparison, including OpenAI (GPT4, GPT o1), Deepseek (Deepseek V3, Deepseek R1), Qwen (Qwen-32 B-Instruct, Qwen-14 B-Instruct), and the joint Deepseek at Qwen model (DeepSeek-R1-Distill-Qwen-32 B, DeepSeek-R1-Distill-Qwen-32 B). These models were used to examine the effectiveness of train-time computation and test-time computation. A MEDEC dataset constructed from a preprint paper provides medical research records on the task of quality control and error correction within EMRs [18]. Based on a partially publicly available MEDEC dataset, our study compares the performance differences between LLMs in medical error detection and correction tasks across multiple dimensions. This study aims to compare the performance and practical effectiveness of LLMs in detecting and correcting errors in EMRs under different computational resource allocation strategies—train-time computation and test-time computation. We further seek to identify the optimal model for train-time computation and test-time computation individually or in assembly.

There is a lack of research on computational resource input for the EMR error correction system in current clinical practice. The purpose of this study is to investigate the ability of train-time computation, test-time computation, and a large language model that combines the two to identify and correct errors in EMR. We hypothesize that the integrated model combining train-time computation and test-time computation will outperform a single large model in EMR error identification and correction, and the analysis will provide an empirical basis for exploring new ways to manage the quality of EMR more efficiently. Ultimately, this may help reduce medical errors and improve the overall quality of medical care.

## 2. Data and Methods

### 2.1. Study Design and Data Source

**Figure 1:**
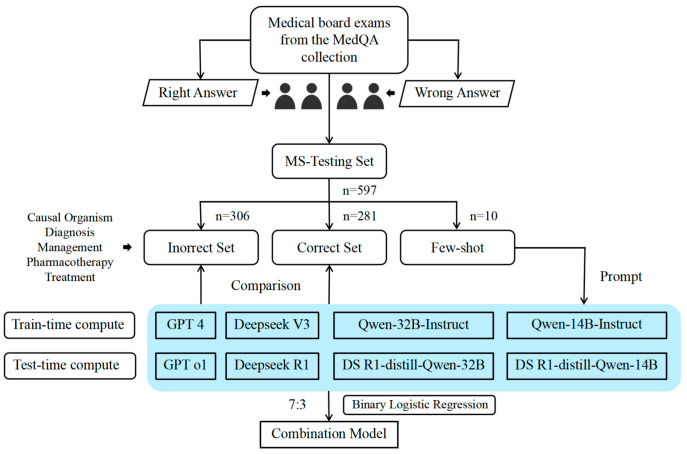
Flow chart of the study (The blue boxes are the large language models chosen for this study).

This study uses the MEDEC-MS dataset (https://github.com/abachaa/MEDEC/tree/main/MEDEC-MS), which was released on 31 December 2024 and accessed on 28 February 2025. MEDEC is the first dataset developed for detecting and correcting medical errors in EMRs [18]. It consists of two datasets, MEDEC-MS and MEDEC-UW, which cover five types of errors: causal organism, diagnosis, management, pharmacotherapy, and treatment [18]. The MEDEC-MS dataset was constructed by four researchers with medical backgrounds. It was developed using medical narratives and multiple-choice questions from the medical board exams in the MedQA collection [19], with errors deliberately introduced into 3360 real-world clinical records, data-driven from real-world scenarios. Each medical record text contains either a correct statement or an error. Since this study is evaluating the ability of LLMs to recognize and correct errors in EMR, only 597 MEDEC-MS validation datasets were selected for the study, and the results obtained were then used for the development and validation of the assembly model. Because the MEDEC-MS validation dataset is public data, only after the LLMs evaluated in this study have completed their large-scale pre-training were to complete their training on the training set. Therefore, the 597 medical record texts were not involved in the training of the LLMs, which effectively avoids the risk of overlapping pre-training data and ensures both the fairness of the evaluation results and the true generalization ability of the models. The dataset included 311 erroneous medical record texts and 286 correct medical record texts. The flow chart is shown in Figure 1. Data use was authorized, and all records were de-identified, exempting the requirement for patient informed consent.

### 2.2. Error Type Definitions

The MEDEC-MS dataset covers five categories of errors in causal organism, diagnosis, management, pharmacotherapy, and treatment: (1) causal organism errors refer to inaccuracies of pathogenic organisms or pathogenic pathogens in the medical record; (2) diagnostic errors refer to inaccuracies of the diagnosis provided in the medical record; (3) management errors refer to inaccurate next medical actions of the physician for the patient in the medical record; (4) pharmacotherapy errors refer to inaccurate medication recommended in the medical record; (5) treatment errors refer to inaccurate treatments recommended in the medical record. During the model training in this study, the models were designed to assess the quality of medical records by detecting errors, annotating errors, and providing corrections based on the five types of errors. Accordingly, the study defines whether the sample is erroneous or not as a binary variable (0: correct, 1: erroneous). The erroneous sentence ID was determined based on the specific location of the sentence in the medical record text. Error types were replaced by 1 to 5, corresponding to causal organism, diagnosis, management, pharmacotherapy, and treatment, respectively. Models were considered correct under two conditions: (1) correctly identifying a medical record without errors, or (2) correctly recognizing an erroneous medical record and predicting both the correct error type and erroneous sentence ID.

### 2.3. Model Selection

We examined the effectiveness of multiple models in error detection and correction of medical record texts. The models selected for comparison included GPT4 and GPT o1 models (Open AI, San Francisco, CA, USA), Deepseek V3 and Deepseek R1 (DeepSeek, Hangzhou, China), Qwen 32 B-Instruct and Qwen 14 B-Instruct (Alibaba, Hangzhou, China), as well as DeepSeek-R1-Distill-Qwen-32B and DeepSeek-R1-Distill-Qwen-32B (DeepSeek and Qwen’s joint models). Model classifications are shown in Table 1, while model versions and parameter sizes are shown in Table S1. These models cover both train-time computation and test-time computation with two different computational resource allocations. They also include both distilled models and models fine-tuned with precise instructions to comprehensively assess the inference efficiency and instruction execution capability [20].

### 2.4. Experimental Methods

Based on the types of errors and textual characteristics of medical record texts, we developed a prompt engineering method (Table S2). Firstly, we used the role definition to narrow the question threshold by instructing it with the phrase: “You are an expert in detecting errors in medicine and medical records”, thereby reducing dichotomous interference. Secondly, the errors of medical records were strictly limited to five aspects: causal organism, diagnosis, management, pharmacotherapy, and treatment. The LLMs were asked to ignore general errors such as sentence morphemes, imprecision, incompleteness, and insufficiency. All medical record texts were partitioned into five error types and contained several pairs of randomly ordered medical record texts (erroneous medical record text and corrected medical record text) within the same error type area. Since the medical record text pairs within the same error type were randomly ordered, we systematically selected the first medical record text pair of each error type, employed random sampling to avoid selection bias, and ensured that all error types were covered (Table 2). Erroneous medical record text and correct medical record text were examples of fixed pairs to avoid manual matching bias. All examples are shown in Table S3. The study successively attempted the four classical methods of ZeroShot, OneShot, FewShot, and Chain-of-Thought for testing. Since GPT 4 performs comparably to a professional human reviewer in the classification of clinical texts [21,22], the study also trained evaluation models based on GPT 4 (the model cue word engineering is shown in Table S2) to evaluate the consistency of the error correction results of the model after anonymization with the error correction results of the standard answer. We defined four scoring levels: A (the corrected answer is 100% consistent with the standard answer), B (the corrected answer is more than 80% consistent with the standard answer), C (the corrected answer is more than 50% consistent with the standard answer), and D (the corrected answer is less than 50% consistent with the standard answer). These correspond to a score of 1, 0.75, 0.5, and 0, respectively. Evaluation models were required to cite the original text to provide a brief 300-word rationale, and Cohen’s Kappa tests for repeated ratings of the same sample one month apart were all greater than 0.9 to ensure the reliability of the ratings (Table S4).

### 2.5. Model Evaluation and Statistical Analysis

The study used precision, recall, and F1 score as evaluation metrics to measure the performance of the models. Since quality control of medical record texts prioritizes the detection of as many errors as possible, precision was the primary focus of this study. When precision could not distinguish model performance, the F1 score was used for further comparison. We also used accuracy to evaluate the performance of LLMs in detecting errors across all error types. We also defined error correction accuracy to score the model’s error correction ability. This metric was calculated by dividing the evaluation score of the evaluated model by the total number of erroneous samples detected by the initial model. In addition, we utilized a binary logistic regression model to construct an optimal combination model by integrating the LLMs that performed well on individual error or all error types. A total of 587 medical record texts were randomly divided into a training set (*n* = 411) and a testing set (*n* = 176) using a random number table method with a ratio of 7:3. Correlation coefficients and variance inflation factors were used to assess high degree of covariance, five-fold cross-validation was used to calculate the area under the curve (AUC) to evaluate the discriminative performance of the combined model, and weight was used to assess the contribution of the LLMs in the assembled model. All statistical analyses were conducted in Python (version 3.9).

## 3. Results

### 3.1. Detection–Correction of Medical Records

Comparative results after training with different prompt engineering methods revealed that under the ZeroShot and OneShot methods, different models showed significant discrepancies in understanding the prompts, leading to a substantial difference in the detection rate. While it is difficult to standardize the performance of the models under the chain-of-thought approach, the FewShot method is more effective than the other methods [23,24]. Therefore, the study finally adopted the FewShot method. The 587 MEDEC-MS test datasets were input into different models, and the error labeling results for medical record texts were obtained. Table 3 uses ms-4 clinical text as an example to present the performance of different models in detecting error types and erroneous sentences, applying corrections to erroneous sentences, and the scores of the evaluation models. It can be observed that the models focusing on test-time computation exhibit longer reasoning time than the models focusing on train-time computation. All models were able to detect errors in medical records and provide correct corrections.

### 3.2. Analysis of Medical Record Error Detection and Correction

**Figure 2:**
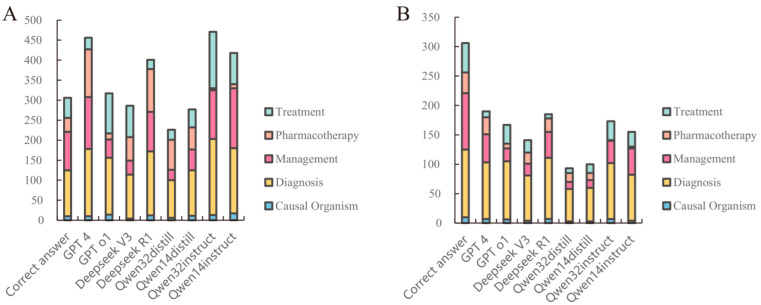
Detecting errors in medical record texts by LLMs ((**A**) is the number of errors detected by LLMs, (**B**) is the number of errors correctly detected by LLMs).

The eight LLMs used in the study analyzed and evaluated a total of 587 medical record texts. The results of error type detection by each type of LLM are shown in Figure 2A, and the results of correct error type detection are shown in Figure 2B. The instruction fine-tuning class model demonstrated stricter error detection, with Qwen-32 B-Instruct detecting the highest number of errors. The distillation inference class models were more lenient in error detection, with DeepSeek-R1-Distill-Qwen-32B and DeepSeek-R1-Distill-Qwen-14B detecting fewer errors. The error types detected by GPT 4 and DeepSeek R1 closely align with the standard answer error types, although they tend to produce more false positives. GPT 4 and Deepseek R1 achieved a higher overall rate of correctness in detecting errors, and the distill inference class of models demonstrated weaker error detection capabilities. Detailed results of error detection for each model are shown in Tables S5 and S6.

### 3.3. Performance of Large Language Models

**Figure 3:**
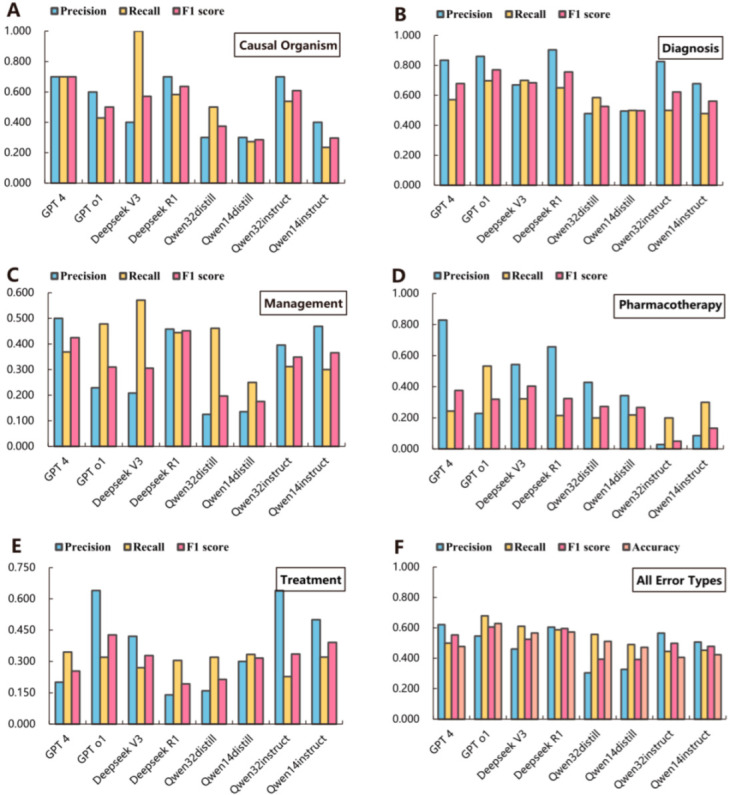
Precision, recall, F1 score, and accuracy of each model in detecting errors ((**A**) is the result of the models analyzing only causal organism error, (**B**) is the result of the models analyzing only diagnosis error, (**C**) is the result of the models analyzing only management errors, (**D**) is the result of the models analyzing only pharmacotherapy error, (**E**) is the result of the models analyzing only treatment error, and (**F**) is the result of the models for overall error types in the medical records).

The medical record texts were analyzed using the LLMs selected for the study. Model performance is shown in Figure 3, with detailed results provided in Table S7. We evaluated the error detection ability of all models across the five types of medical record text errors, and calculated precision, recall, and F1 scores for each error type, as well as for all error types combined (Figure 3A–E). In addition, we calculated the accuracy across all error types (Figure 3F). The results showed that the GPT-4 model achieved the highest precision for causal organism errors (0.700, 95%CI: 0.416–0.984), management errors (0.500, 95%CI: 0.400–0.600), and pharmacotherapy errors (0.829, 95%CI: 0.704–0.953); DeepSeek R1 achieved the highest precision for diagnosis errors (0.904, 95%CI: 0.851–0.958); and GPT-o1 achieved the highest precision for treatment errors (0.640, 95%CI: 0.507–0.773). Against a general model, GPT 4 demonstrated higher precision but was less accurate than GPT o1 (precision: 0.621 vs. 0.546, accuracy: 0.477 vs. 0.629). Deepseek R1 outperformed Deepseek V3 in error detection on medical record text (precision: 0.605 vs. 0.461, accuracy: 0.572 vs. 0.566). The instruction fine-tuning models (focusing on train-time computation) outperformed the distillation inference models (focusing on test-time computation) in terms of precision but exhibited lower accuracy. But both types of models demonstrated poor performance.

### 3.4. Error Correction Accuracy of Large Language Models

**Figure 4:**
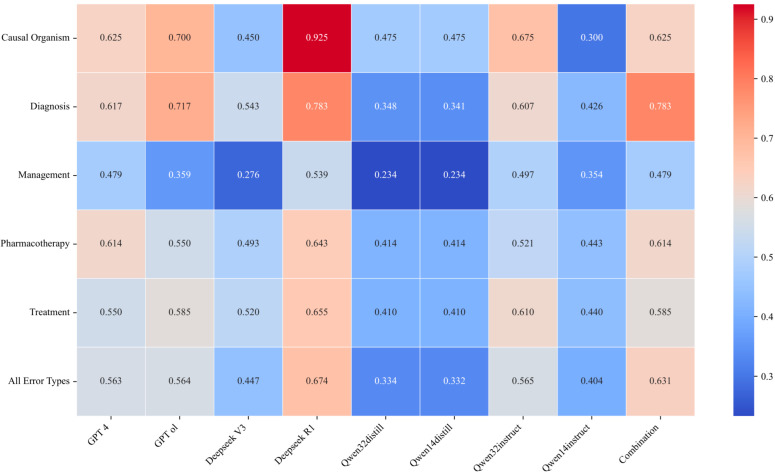
Heat map of the error correction accuracy of the models (the darker the red color, the better the error correction accuracy of the model; the darker the blue color, the worse the error correction accuracy of the model; and a total score of “1” is the full score of error correction accuracy).

The study also utilized the GPT-4 training evaluation model to assess the error correction capability of all models. A heat map of the accuracy is shown in Figure 4. The results showed that GPT o1 and Deepseek R1 models, which focus on test-time computation, achieved higher error correction accuracy compared to GPT 4 and Deepseek V3 models, which focus on train-time computation. Qwen-32 B-Instruct model exhibited relatively high error correction accuracy, but the rest of the distillation inference class models and instruction fine-tuning class models performed poorly in error correction accuracy in medical record text.

### 3.5. Assembly of Large Language Models

**Figure 5:**
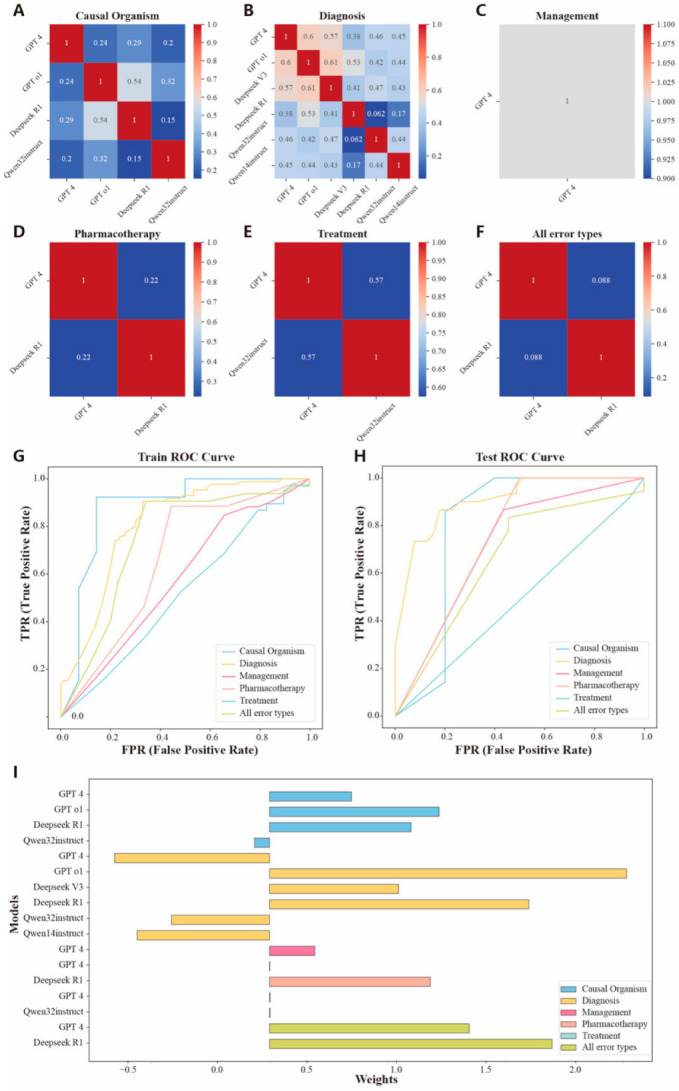
Effectiveness evaluation of the assembly model ((**A**) is the model correlation analysis in causal organism error, and “1” means perfect positive correlation; (**B**) is the model correlation analysis in diagnosis error, and “1” means perfect positive correlation; (**C**) is the model correlation analysis in management error, and “1” means perfect positive correlation; (**D**) is the model correlation analysis in pharmacotherapy error, and “1” means perfect positive correlation; (**E**) is the model correlation analysis in treatment error, and “1” means perfect positive correlation; (**F**) is model correlation analysis in combining all error types, and “1” means perfect positive correlation; (**G**) is the ROC curve of assembly models in the training set for each error type and all error types; (**H**) is the ROC curve of assembly models in the testing set for each error type and all error types; (**I**) is the weight distribution of individual models in the assembly model).

We selected LLMs with a precision greater than 0.6 for each error type and performed an assembled analysis of multiple LLMs using a binary logistic regression to construct the optimal assembly of models. Since both train-time and test-time computation-focused LLMs achieved a precision score below 0.6 for the management error type, the best-performing model, GPT-4, was selected as the final model for this category. Before the model assembly analysis, we performed a correlation analysis to exclude highly correlated models. The matrix of correlation coefficients and the types of LLMs selected for each error type are shown in Figure 5A–F. We also calculated variance inflation factors, all less than 5, which were used to detect multicollinearity between models (Table S8). Next, the large language models screened for each type of error were assembled and analyzed using five-fold cross-validation, with the standardized answers of the medical record text as the dependent variable. The performances of the assembly model on the training and testing sets for each error type and all error types combined are shown in Figure 5G,H. The results for the assembly model for individual and all error types are summarized as follows: For causal organism error, the AUC was 0.879 (0.742–1.000, *p* < 0.001) on the training set and 0.800 (0.542–1.000, *p* = 0.045) on the testing set. For diagnosis error, the AUC was 0.810 (0.743–0.876, *p* < 0.001) on the training set and 0.908 (0.831–0.985, *p* < 0.001) on the testing set. For the management error, the AUC was 0.578 (0.489–0.666, *p* = 0.045) on the training set and 0.715 (0.591–0.840, *p* < 0.001) on the testing set. For the pharmacotherapy error, the AUC was 0.661 (0.500–0.822, *p* = 0.036) on the training set and 0.750 (0.523–0.977, *p* = 0.009) on the testing set. For the treatment error, the AUC was 0.520 (0.380–0.660, *p* = 0.392) on the training set and 0.488 (0.271–0.704, *p* = 0.619) on the testing set. For all error types, the AUC was 0.745 (0.625–0.864, *p* < 0.001) on the training set and 0.662 (0.461–0.862, *p* = 0.045) on the testing set. The performances of the individual LLMs and the assembled models are shown in Table 4. In addition, we analyzed the weight distribution of the LLMs within the assembly model. The distribution of model weights is shown in Figure 5I. The GPT o1 model demonstrated significant advantages in causal organism and diagnosis error types, while the Deepseek R1 model has strong weights in the pharmacotherapy error type and the overall analysis across all error types. The GPT-4 model exhibited strong discriminative capability in the assembly model for management and treatment error types. The Qwen series of instruction fine-tuning class model demonstrated negative weights in the assembly model, suggesting a potential tendency toward bias in the textual error detection of medical records.

## 4. Discussion

We selected four LLMs focusing on train-time computation and four LLMs focusing on test-time computation to detect and correct errors in real-world medical record texts. This study builds on the MEDEC dataset developed by Asma Ben Abacha et al., which supports the evaluation of popular LLMs for medical record text error detection and correction [18]. It specifically explores the performance of LLMs, focusing on train-time computation and focusing on test-time computation in the context of medical record errors detection and corrections. Using the prompt engineering of the FewShot method, we selected and trained a total of 8 LLMs, including non-inference models, inference models, distilled inference class models, and instruction fine-tuning class models. In addition, we developed prompt engineering and designed a 4-level scoring evaluation model to assess the models’ error correction ability. To the best of our knowledge, this is the first study that explores the performance of LLMs focusing on train-time computation and focusing on test-time computation in detecting and correcting errors in medical record texts, rather than focusing solely on individual model performance comparisons. Despite the substantial progress made in LLMs, the reasoning accuracy of these information technologies remains far below human-level performance. The performance evaluation of LLMs under different computational resource allocation strategies provides valuable insights for generative artificial intelligence-enabled quality control of EMRs.

Although the errors in medical record texts used in the study were artificially introduced based on medical board exam materials, errors in real-world electronic medical records can be more complex and varied. However, the medical record texts were derived from real-world data, providing a reliably representative and strongly relevant research. Our results show that models focusing on train-time computation exhibited shorter reasoning time, stricter error detection behavior, and relatively higher accuracy in detecting errors. Non-inference-based models outperform inference-based models in terms of task processing speed due to the absence of the inference process. In addition, the GPT-4 model was able to more accurately capture errors in the medical record text, which may be attributed to the substantial computational resources invested during its training phase [25]. This is consistent with the findings of Gertz R.J. et al. [26], who reported that the GPT-4 model performs well in detecting errors in radiology reports. The study by Geert Litjens et al. showed that increasing computational resources can significantly improve the model performance in anomaly detection tasks by enhancing the ability to extract contextual information [27]. GPT-4 is a model that focuses on train-time computation, following a skewed distribution strategy that allocates intensive computational resources during the pre-training phase. This approach strengthens its capability to detect semantic anomalies. In the task of medical record text error detection, models focusing on test-time computation performed significantly worse than those focusing on train-time computation. The overall performance of the test-time computation model may still be affected by model illusion and limitations in knowledge accuracy. This suggests that allocating computational resources toward the training phase is essential for effective application of LLMs in textual anomaly detection within medical records. In addition, we found that distillation-like inference models offered no clear advantage compared to models with a similar scale.

In this study, where we detected that models may vary in their ability to detect different error types, we evaluated the models’ performance separately for each of the five error types in medical record texts. Our results highlight that different error types require different modeling capabilities. The GPT-4 model performed best in detecting causal organism, management, and pharmacotherapy errors, while the models focusing on test-time computation (Deepseek R1 and GPT o1) achieved better results in detecting diagnosis and treatment errors. A possible explanation is that error detection in the first three error types requires less inference and relies more heavily on the structured knowledge acquired from the pre-training phase. Massive amounts of data and computational power in the training phase give the GPT-4 model (focusing on train-time computation) a notable performance advantage. GPT 4 has a relatively large number of parameters and a complex transformer architecture, allowing it to capture a wider and deeper range of knowledge and capabilities during the pre-training phase [28]. This implies that the GPT 4 model may be able to better understand medical texts dealing with relevant content involving causality, managerial decisions, and complex pharmacological information through more powerful contextual modeling capabilities and a richer implicit knowledge base [29,30].

Diagnosis and treatment error types usually involve reasoning and judgment of medical knowledge, which requires the model to engage in a deeper reasoning process.

In contrast, models focusing on test-time computation may be more dominant in the reasoning process because of the ability of these models to perform additional computation, chain thinking, or self-correction during the reasoning phase [31,32]. Models focusing on test-time computation often dynamically learn and adapt behaviors to better process, analyze, and integrate complex healthcare relationships through multistep reasoning, retrieval enhancements, or specific command-following mechanisms [12]. For example, during the prompt engineering, Schistosoma mansoni infection was incorrectly diagnosed as hepatitis A in the ms-test-0, and azithromycin was incorrectly treated as vancomycin in the ms-test-503. This requires the model to perform a reinforcement learning inference task to understand the prompt engineering content and perform contextual inference, which helps minimize interpretation errors in the medical record text. To further evaluate the model’s performance, we trained the evaluation model based on GPT-4 to assess the consistency between the standard answer and the model-corrected answer. Deepseek R1 and GPT o1 demonstrated higher error correction accuracy, which may be attributed to the nature of the corrected tasks requiring holistic inference and judgment across the entire medical record [33,34]. These tasks are better handled by the inference class of the model, focusing on test-time computation.

Based on this, we selected the models with a threshold of precision greater than 0.6 and utilized a logistic regression model for the assembly model. Our results found a relatively significant improvement in the performance of the combined model in terms of error identification and correction for all error types combined (assembled model: precision: 0.737, *p* < 0.05). Although further improvement is needed in some error types, these limitations may be attributed to model selection or sample size constraints. Visualization of individual models’ contribution to the assembly model revealed that models focusing on train-time computation and models focusing on test-time computation exhibited different influences on the assembly model, targeting different error types. This suggests that assembly models may lead to greater performance gains. With the continued advancement of LLMs, computational resource allocation is gradually shifting from train-time computation to test-time computation. Test-time computation focuses more on inference rather than matching in the model knowledge learning process. Its inference effect is more obvious and provides an overwhelming advantage over non-inference task models. However, investing computational resources in either train-time computation or test-time computation LLMs presents certain drawbacks. Models focusing on train-time computation aim to build powerful and extensive knowledge bases, and the parameter sizes of the models have been expanded to hundreds of billions. This approach requires large-scale computational resources during the model training phase, resulting in significantly high costs. In addition, investing computational resources in the training modeling phase also poses the threat of diminishing returns over time. Shifting computational resources to the model inference phase may continue to improve model performance, even when the gains in the model training phase are limited. Models focusing on test-time computation aim to use knowledge and computational resources in the inference process [35]. However, test-time resource allocation prolongs processing time and increases arithmetic power, which may impose ongoing operational costs. For tasks that require more complex reasoning, using models that focus on test-time computation may yield greater overall benefit. Therefore, we propose that assembling train-time computation models with strong knowledge bases with test-time computation models that excel at reasoned thinking can create combined models that ensure both high accuracy and high efficiency. Such integration is likely to optimize model performance and further advance medical artificial intelligence.

This study also has some limitations. The study only used partial models focusing on train-time computation and test-time computation. Although this study included distillation inference class models and instruction fine-tuning class models as supplements, future studies could still incorporate more diverse models for comparison. Despite the fact that this study used a small sample size of single-center medical record texts and all medical record errors were adapted from American medical board exam questions, the types of medical record errors and cross-cultural medical care were limited. However, we believe the results still hold meaningful research value and potential for generalization. Further studies could consider building real-world multicenter medical record text datasets to enable more comprehensive and in-depth investigations. In addition, although the medical record errors in the dataset were annotated by professionals, their reliability needs to be further explored.

## 5. Conclusions

This study applies models focusing on train-time computation, test-time computation, and their assembly to the detection and correction of errors in medical record texts. The findings and insights from this study contribute to the application of LLMs in error detection and quality management of EMRs. The exploration and application of LLMs in EMRs further promote rapid improvement in medical quality and the accelerated development of medical information.

## Figures and Tables

**Table 1:** Classification of the models used in the study.

Models	Computing Resources
GPT 4	Focusing on Train-time Computation
GPT o1	Focusing on Test-time Computation
DeepSeek V3	Focusing on Train-time Computation
DeepSeek R1	Focusing on Test-time Computation
DeepSeek-R1-Distill-Qwen-32B	Focusing on Test-time Computation
DeepSeek-R1-Distill-Qwen-14B	Focusing on Test-time Computation
Qwen-32B-Instruct	Focusing on Train-time Computation
Qwen-14B-Instruct	Focusing on Train-time Computation

**Table 2:** Example of model learning erroneous medical record text, 5 cases.

Medical Record ID	Medical Records	Error Type	Error Sentence ID	Error Sentence	Corrected Sentences
ms-0	A 29-year-old internal medicine resident presents to the emergency department with complaints of fever, diarrhea, abdominal pain, and skin rash for 2 days. He feels fatigued and has lost his appetite. On further questioning, he says that he returned from his missionary trip to Brazil last week. He is excited as he talks about his trip. Besides a worthy clinical experience, he also enjoyed local outdoor activities, like swimming and rafting. His past medical history is insignificant. The blood pressure is 120/70 mm Hg, the pulse is 100/min, and the temperature is 38.3 °C (100.9 F). On examination, there is a rash on the legs. The patient’s symptoms are suspected to be due to hepatitis A. The rest of the examination is normal.	Causal Organism	10	Patient’s symptoms are suspected to be due to hepatitis A.	Patient’s symptoms are suspected to be due to Schistosoma mansoni.
ms-21	A 3-year-old Cuban-American male has a history of recurrent Pseudomonas and Candida infections. Laboratory analysis reveals no electrolyte abnormalities. Examination of his serum shows decreased levels of IgG and CT scan reveals the absence of a thymus. The child likely has common variable immunodeficiency.	Diagnosis	3	The child likely has common variable immunodeficiency.	The child likely has severe combined immunodeficiency syndrome.
ms-281	A 57-year-old man with a known angina pectoris starts to experience a severe burning retrosternal pain that radiates to his left hand. After 2 consecutive doses of sublingual nitroglycerin taken 5 min apart, there is no improvement in his symptoms, and the patient calls an ambulance. Emergency medical service arrives within 10 min and begins evaluation and prehospital management. The vital signs include the following: blood pressure 85/50 mm Hg, heart rate is 96/min, respiratory rate is 19/min, temperature is 37.1 °C (98.9 °F), and SpO2 89% in ambient air. Oxygen supply and intravenous access are established. Aspirin 81 mg is administered, and the patient is transported to a percutaneous coronary intervention center after an ECG shows the findings in the given image.	Management	8	Aspirin 81 mg is administered, and the patient is transported to a percutaneous coronary intervention center after an ECG shows the findings in the given image.	Aspirin 325 mg is administered, and the patient is transported to a percutaneous coronary intervention center after an ECG shows the findings in the given image.
ms-472	A 65-year-old man is hospitalized after undergoing operative fixation of a left distal radius fracture due to a fall. On postoperative day 1, he reports having several episodes of palpitations with associated lightheadedness. He denies any chest pain and states that these episodes last for a few seconds each before resolving. On examination, his temperature is 98.4 °F (36.9 °C), blood pressure is 124/76 mmHg, pulse is 94/min, and respirations are 12/min. The patient is currently asymptomatic without palpitations. The patient is immediately connected to a cardiac monitor which shows normal sinus rhythm. Over the course of the night, the monitor demonstrates 3 more episodes of the rhythm shown below in the Figure, along with the associated palpitations and lightheadedness. Blood pressure and oxygen saturation are stable during these episodes. The patient is given intravenous magnesium and diltiazem after the decision is made to also start medical therapy.	Pharmacotherapy	9	The patient is given intravenous magnesium and diltiazem after the decision is made to also start medical therapy.	The patient is given intravenous magnesium and amiodarone after the decision is made to also start medical therapy.
ms-503	A 14-year-old female notes that while her fever, malaise, and a sore throat have dissipated a week ago, her hacking cough persists. A chest radiograph (Figure) demonstrates a streaky infiltrate in her right lung that appears much worse than the symptoms she is experiencing. The patient is given vancomycin after a cold agglutination test conducted on her blood shows clumping together when placed at 4 degrees Celsius.	Treatment	2	The patient is given vancomycin after a cold agglutination test conducted on her blood shows clumping together when placed at 4 degrees Celsius.	The patient is given azithromycin after a cold agglutination test conducted on her blood shows clumping together when placed at 4 degrees Celsius.

**Table 3:** Error detection, annotation, and correction results of LLMs for the ms-4 medical record (Error type: Causal Organism).

Sample/Models	Thinking Time	Sentence ID	Error Sentence	Corrected Sentence	Score by GPT 4
ms-4	/	3	The causative agent is an irregular, non-septate yeast with wide angles.	The causative agent is a commensal yeast that is catalase-positive.	/
ChatGPT-4	6.62 s	3	The causative agent is an irregular, non-septate yeast with wide angles.	The causative agent is a regular, septate fungus with branching at narrow angles.	C
ChatGPT o1	13.58 s	3	The causative agent is an irregular, non-septate yeast with wide angles.	The causative agent is a budding yeast with pseudohyphae, consistent with Candida species.	B
Deepseek V3	8.93 s	3	The causative agent is an irregular, non-septate yeast with wide angles.	The causative agent is Candida albicans, a yeast that typically causes intertrigo in skin folds.	C
Deepseek R1	17.40 s	3	The causative agent is an irregular, non-septate yeast with wide angles.	The causative agent is an oval-shaped budding yeast with pseudohyphae (Candida species).	B
Deepseek R1 -Distill-Qwen-32 B	15.14 s	3	The causative agent is an irregular, non-septate yeast with wide angles.	The causative agent is a Candida species, which is a round, septate yeast.	B
Deepseek R1 -Distill-Qwen-14B	19.59 s	3	The causative agent is an irregular, non-septate yeast with wide angles.	The causative agent is an irregular, non-septate yeast, likely Candida species.	B
Qwen-32B-Instruct	5.89 s	3	The causative agent is an irregular, non-septate yeast with wide angles.	The causative agent is a Candida species, which is a common yeast with irregular, non-septate hyphae.	B
Qwen-14B-Instruct	4.25 s	3	The causative agent is an irregular, non-septate yeast with wide angles.	The causative agent is Candida, a common yeast that can cause intertrigo in obese individuals.	B

“score by GPT 4” means the score of the evaluation model based on the GPT 4 model for the performance of each of the large language models.

**Table 4:** Performance of individual LLMs and assembled models on different error types.

Error Types	Causal Organism	Diagnosis	Management	Pharmacotherapy	Treatment	All Error Types
Best individual LLMs	GPT-4	Deepseek R1	GPT-4	GPT-4	GPT o1	GPT-4
Precision	0.700 (0.416, 0.984)	0.904 (0.851, 0.958) ***	0.500 (0.400, 0.600)	0.829 (0.704, 0.953) ***	0.640 (0.507, 0.773)	0.621 (0.567, 0.675) ***
Recall	0.700 (0.416, 0.984)	0.650 (0.576, 0.724) ***	0.369 (0.286, 0.452) **	0.244 (0.167, 0.321) ***	0.320 (0.229, 0.411) ***	0.499 (0.449, 0.549)
F1 score	0.700 (0.499, 0.901)	0.756(0.703, 0.810)	0.425 (0.359, 0.491)	0.377 (0.284, 0.470)	0.427 (0.340, 0.513)	0.553 (0.516, 0.591)
Accuracy	\	\	\	\	\	0.477 (0.437, 0.517)
Assembly Models						
Precision	0.857 (0.598, 1.000) *	0.788 (0.648, 0.927) ***	0.605 (0.459, 0.751)	0.643 (0.392, 0.894)	0.414 (0.235, 0.593)	0.737 (0.539, 0.935) *
Recall	0.857 (0.598, 1.000) *	0.867(0.745, 0.988) ***	0.867 (0.745, 0.988) ***	1.000 (1.000, 1.000) **	1.000 (1.000, 1.000) **	0.778 (0.586, 0.979) *
F1 score	0.857 (0.659, 1.000)	0.825 (0.736, 0.915)	0.712 (0.606, 0.819)	0.783 (0.597, 0.968)	0.585 (0.406, 0.765)	0.757 (0.601, 0.913)
Accuracy	0.833 (0.622, 1.000) *	0.841 (0.754, 0.927) ***	0.696 (0.587, 0.804) ***	0.737 (0.539, 0.935) *	0.414 (0.235, 0.593)	0.690 (0.521, 0.858) *

* *p* < 0.05, ** *p* < 0.01, *** *p* < 0.001 (two-tailed tests).

## Data Availability

The original data presented in the study are openly available in Github at https://github.com/abachaa/MEDEC/tree/main/MEDEC-MS (accessed on 28 February 2025).

## Supplementary Materials

The following supporting information can be downloaded at: https://www.mdpi.com/article/10.3390/diagnostics15141829/s1. Table S1: Versions and parameter sizes of the large language models used in the study. Table S2: Prompt Engineering. Table S3: FewShot list. Table S4: Cohen’s Kappa Test for the evaluation of eight large language models based on the evaluation model of GPT 4. Table S5: Number of errors in medical records detected by eight large language models. Table S6: Number of erroneous and correct medical records detected by eight large language models. Table S7: Model Precision, Recall, F1 Score, Accuracy for eight large language models in different error types. Table S8: Variance inflation factors for assembled large language models. Table S9: Hyper-parameter tuning for logistic regression models.

## Abbreviations

The following abbreviations are used in this manuscript:

EMRs	Electronic medical records
LLMs	Large language models
AUC	Area under the curve

## References

[1] Persaud N. (2019). A national electronic health record for primary care. CMAJ.

[2] Steinkamp J., Kantrowitz J.J., Airan-Javia S. (2022). Prevalence and sources of duplicate information in the electronic medical record. JAMA Netw. Open.

[3] Nijor S., Rallis G., Lad N., Gokcen E. (2022). Patient safety issues from information overload in electronic medical records. J. Patient Saf..

[4] Cao Z., Keloth V.K., Xie Q., Qian L., Liu Y., Wang Y., Shi R., Zhou W., Yang G., Zhang J. (2025). The Development Landscape of Large Language Models for Biomedical Applications. Annu. Rev. Biomed. Data Sci..

[5] Li L., Zhou J., Gao Z., Hua W., Fan L., Yu H., Hagen L., Zhang Y., Assimes T.L., Hemphill L. (2024). A scoping review of using large language models (LLMs) to investigate electronic health records (EHRs). arXiv.

[6] Yu W., Xiong L., Feifei Z., Jiayu L., Shaoyong C. (2023). Application of Medical Record Quality Control System Based on Artificial Intelligence. J. Sichuan Univ. (Med. Sci.).

[7] Omar M., Ullanat V., Loda M., Marchionni L., Umeton R. (2024). ChatGPT for digital pathology research. Lancet Digit. Health.

[8] Annevirta J. (2025). Intelligent Patient Safety Incident Reporting–Process Design and Feasibility of Utilizing LLM for Report Generation. Master’s Thesis.

[9] Yang X., Chen A., PourNejatian N., Shin H.C., Smith K.E., Parisien C., Compas C., Martin C., Costa A.B., Flores M.G. (2022). A large language model for electronic health records. NPJ Digit. Med..

[10] Shi W., Xu R., Zhuang Y., Yu Y., Zhang J., Wu H., Zhu Y., Ho J., Yang C., Wang M.D. (2024). Ehragent: Code empowers large language models for few-shot complex tabular reasoning on electronic health records. arXiv.

[11] Menezes M.C.S., Hoffmann A.F., Tan A.L., Nalbandyan M., Omenn G.S., Mazzotti D.R., Hernández-Arango A., Visweswaran S., Venkatesh S., Mandl K.D. (2025). The potential of Generative Pre-trained Transformer 4 (GPT-4) to analyse medical notes in three different languages: A retrospective model-evaluation study. Lancet Digit. Health.

[12] Xu F., Hao Q., Zong Z., Wang J., Zhang Y., Wang J., Lan X., Gong J., Ouyang T., Meng F. (2025). Towards Large Reasoning Models: A Survey of Reinforced Reasoning with Large Language Models. arXiv.

[13] Radford A., Narasimhan K., Salimans T., Sutskever I. (2018). IMPROVING language Understanding by Generative Pre-Training. https://api.semanticscholar.org/CorpusID:49313245.

[14] Peng B., Chena K., Zhangc Y., Wangd T., Bie Z., Yif H., Songg X., Zhanga C., Liuh M., Liang C.X. (2025). Optimizing the Last Mile: Test-Time Compute Strategies for Next-Generation Language Models. Preprint.

[15] Snell C., Lee J., Xu K., Kumar A. (2024). Scaling llm test-time compute optimally can be more effective than scaling model parameters. arXiv.

[16] Pitis S., Zhang M.R., Wang A., Ba J. (2023). Boosted prompt ensembles for large language models. arXiv.

[17] Matarazzo A., Torlone R. (2025). A Survey on Large Language Models with some Insights on their Capabilities and Limitations. arXiv.

[18] Abacha A.B., Yim W.-w., Fu Y., Sun Z., Yetisgen M., Xia F., Lin T. (2024). Medec: A benchmark for medical error detection and correction in clinical notes. arXiv.

[19] Jin D., Pan E., Oufattole N., Weng W.-H., Fang H., Szolovits P. (2021). What disease does this patient have? A large-scale open domain question answering dataset from medical exams. Appl. Sci..

[20] Mamunoor R. (2023). New Mixture of Distillation Strategy for Knowledge Transfer. Master’s Thesis.

[21] Wang Y., Huang Y., Nimma I.R., Pang S., Pang M., Cui T., Kumbhari V. (2024). Validation of GPT-4 for clinical event classification: A comparative analysis with ICD codes and human reviewers. J. Gastroenterol. Hepatol..

[22] Chen S., Li Y., Lu S., Van H., Aerts H.J., Savova G.K., Bitterman D.S. (2024). Evaluating the ChatGPT family of models for biomedical reasoning and classification. J. Am. Med. Inform. Assoc..

[23] Ntinopoulos V., Biefer H.R.C., Tudorache I., Papadopoulos N., Odavic D., Risteski P., Haeussler A., Dzemali O. (2025). Large language models for data extraction from unstructured and semi-structured electronic health records: A multiple model performance evaluation. BMJ Health Care Inform..

[24] Brown T., Mann B., Ryder N., Subbiah M., Kaplan J.D., Dhariwal P., Neelakantan A., Shyam P., Sastry G., Askell A. (2020). Language models are few-shot learners. Adv. Neural Inf. Process. Syst..

[25] Achiam J., Adler S., Agarwal S., Ahmad L., Akkaya I., Aleman F.L., Almeida D., Altenschmidt J., Altman S., Anadkat S. (2023). Gpt-4 technical report. arXiv.

[26] Gertz R.J., Dratsch T., Bunck A.C., Lennartz S., Iuga A.-I., Hellmich M.G., Persigehl T., Pennig L., Gietzen C.H., Fervers P. (2024). Potential of GPT-4 for detecting errors in radiology reports: Implications for reporting accuracy. Radiology.

[27] Litjens G., Kooi T., Bejnordi B.E., Setio A.A.A., Ciompi F., Ghafoorian M., Van Der Laak J.A., Van Ginneken B., Sánchez C.I. (2017). A survey on deep learning in medical image analysis. Med. Image Anal..

[28] Hadi M.U., Qureshi R., Shah A., Irfan M., Zafar A., Shaikh M.B., Akhtar N., Wu J., Mirjalili S. (2023). Large language models: A comprehensive survey of its applications, challenges, limitations, and future prospects. Authorea Prepr..

[29] Gopalakrishnan S., Garbayo L., Zadrozny W. (2024). Causality extraction from medical text using large language models (llms). Information.

[30] Kiciman E., Ness R., Sharma A., Tan C. (2023). Causal reasoning and large language models: Opening a new frontier for causality. arXiv.

[31] Ji Y., Li J., Ye H., Wu K., Xu J., Mo L., Zhang M. (2025). Test-time Computing: From System-1 Thinking to System-2 Thinking. arXiv.

[32] Liu C., Wu J., Wu W., Chen X., Lin L., Zheng W.-S. (2025). Chain of Methodologies: Scaling Test Time Computation without Training. arXiv.

[33] Chen Q., Qin L., Liu J., Peng D., Guan J., Wang P., Hu M., Zhou Y., Gao T., Che W. (2025). Towards reasoning era: A survey of long chain-of-thought for reasoning large language models. arXiv.

[34] Li Z.-Z., Zhang D., Zhang M.-L., Zhang J., Liu Z., Yao Y., Xu H., Zheng J., Wang P.-J., Chen X. (2025). From system 1 to system 2: A survey of reasoning large language models. arXiv.

[35] Zhang Q., Lyu F., Sun Z., Wang L., Zhang W., Hua W., Wu H., Guo Z., Wang Y., Muennighoff N. (2025). A Survey on Test-Time Scaling in Large Language Models: What, How, Where, and How Well?. arXiv.

