# Supplementary material for: Train-Time and Test-Time Computation in Large Language Models for Error Detection and Correction in Electronic Medical Records: A Retrospective Study
Source: Diagnostics (Basel). 2025 Jul 21;15(14):1829. doi: 10.3390/diagnostics15141829 (PMC12293163; doi:10.3390/diagnostics15141829)
Supplement: Supplementary file 1 [file diagnostics-15-01829-s001.zip › diagnostics-3694502-supplementary.pdf]

Supplementary material

Table S1 Versions and parameter sizes of large language models used in the study

Model	Version	Parameter
GPT 4	v4	1800b
GPT o1	v1	300b
DeepSeek V3	v3	761b
DeepSeek R1	v3	761b
DeepSeek-R1-Distill-Qwen-32B	v2.5	32b
DeepSeek-R1-Distill-Qwen-14B	v2.5	14b
Qwen-32B-Instruct	v2.5	32b
Qwen-14B-Instruct	v2.5	14b

Table S2 Prompt Engineering

Model	Prompt
Training Model	You are an expert in the field of medicine and medical record error detection, the material is a real medical record and split by sentence, please determine whether there are sentences with serious errors in **causalOrganism, diagnosis, management, harmacotherapy, treatment,** etc. in the split sentences. If there are, identify the 1 most serious and correct it.<br><br>Definition of a serious error:<br>- Must be a very serious error, not a general flaw;<br>- Ignore any errors in the general sense of the word, such as speech defects, imprecision of expression, lack of rigor, incompleteness, insufficiency, etc;<br>- The errors to be found are strictly limited to the five areas of **causalOrganism, diagnosis, management, harmacotherapy, and treatment.**
Evaluation Model	- There is an error-ridden test chart to test the error-correction capabilities of the large language model, with standardized answers.<br>- The large language model has detected the incorrect sentence in the test chart and provided the corrected answer.<br>- You are asked to make the following choices and provide a brief 300-word justification for the consistency of the large language model's corrected answers with the standard answers:<br>A. The corrected answer is 100% consistent with the standard answer (1 point).

	B. the corrected answer is more than 80% consistent with the standard answer (0.75 points).<br>C. the corrected answer is more than 50% consistent with the standard answer (0.5 points).<br>D. the corrected answer is less than 50% consistent with the standard answer (0 points).

Table S3 Few Shot List

Medical Record ID	Medical Records	Error medical records	Error Type	Error Sentence ID	Error Sentence	Corrected Sentences
ms-0	A 29-year-old internal medicine resident presents to the emergency department with complaints of fevers, diarrhea, abdominal pain, and skin rash for 2 days. He feels fatigued and has lost his appetite. On further questioning, he says that he returned from his missionary trip to Brazil last week. He is excited as he talks about his trip. Besides a worthy clinical experience, he also enjoyed local outdoor activities, like swimming and rafting. His past medical history is insignificant. The blood pressure is 120/70 mm Hg, the pulse is 100/min, and the temperature is 38.3 C (100.9 F). On examination, there is a rash on the legs. Patient's symptoms are suspected to be due to hepatitis A. The rest of the examination is normal.	Yes	Causal Organism	10	Patient's symptoms are suspected to be due to hepatitis A.	Patient's symptoms are suspected to be due to Schistosoma mansoni.
ms-1	A 29-year-old internal medicine resident presents to the emergency department with complaints of fevers, diarrhea, abdominal pain, and skin rash for 2 days. He feels fatigued and has lost his appetite. On further questioning, he says that he returned from his missionary trip to Brazil last week. He is excited as he talks about his trip. Besides a worthy clinical experience, he also enjoyed local outdoor activities, like swimming and rafting. His past medical history is insignificant. The blood pressure is 120/70 mm Hg, the pulse is 100/min, and the temperature is 38.3 C (100.9 F). On examination, there is a rash on the legs. The rest of the examination is normal. Patient's symptoms are suspected to be due to Schistosoma mansoni.	No	NA	NA	NA	NA
ms-21	A 3-year-old Cuban-American male has a history of recurrent Pseudomonas and Candida infections. Laboratory analysis reveals no electrolyte abnormalities. Examination of his serum shows decreased levels of IgG and CT scan reveals the absence of a thymus. The child likely has common variable immunodeficiency.	Yes	Diagnosis	3	The child likely has common variable immunodeficie ncy.	The child likely has severe combined immunodeficie ncy syndrome.
ms-22	A 3-year-old Cuban-American male has a history of recurrent Pseudomonas and Candida infections. Laboratory analysis reveals no electrolyte abnormalities. The child is suspected to have severe combined immunodeficiency syndrome after examination of his serum shows decreased levels of IgG and CT scan reveals the absence of a thymus.	No	NA	NA	NA	NA

ms-281	A 57-year-old man with a known angina pectoris starts to experience a severe burning retrosternal pain that radiates to his left hand. After 2 consecutive doses of sublingual nitroglycerin taken 5 minutes apart, there is no improvement in his symptoms, and the patient calls an ambulance. Emergency medical service arrives within 10 minutes and begins evaluation and prehospital management. The vital signs include: blood pressure 85/50 mm Hg, heart rate 96/min, respiratory rate 19/min, temperature 37.1 C (98.9 F), and SpO2 89% on ambient air. Oxygen supply and intravenous access are established. Aspirin 81 mg is administered and patient is transported to a percutaneous coronary intervention center after an ECG shows the findings in the given image.	Yes	Management	8	Aspirin 81 mg is administered and patient is transported to a percutaneous coronary intervention center after an ECG shows the findings in the given image.	Aspirin 325 mg is administered and patient is transported to a percutaneous coronary intervention center after an ECG shows the findings in the given image.
ms-282	A 57-year-old man with a known angina pectoris starts to experience a severe burning retrosternal pain that radiates to his left hand. After 2 consecutive doses of sublingual nitroglycerin taken 5 minutes apart, there is no improvement in his symptoms, and the patient calls an ambulance. Emergency medical service arrives within 10 minutes and begins evaluation and prehospital management. The vital signs include: blood pressure 85/50 mm Hg, heart rate 96/min, respiratory rate 19/min, temperature 37.1 C (98.9 F), and SpO2 89% on ambient air. Oxygen supply and intravenous access are established. An ECG shows the findings in the given image. Aspirin 325 mg is administered and patient is transported to a percutaneous coronary intervention center.	No	NA	NA	NA	NA
ms-472	A 65-year-old man is hospitalized after undergoing operative fixation of a left distal radius fracture due to a fall. On postoperative day 1, he reports having several episodes of palpitations with associated lightheadedness. He denies any chest pain and states that these episodes last for a few seconds each before resolving. On exam, his temperature is 98.4 F (36.9 C), blood pressure is 124/76 mmHg, pulse is 94/min, and respirations are 12/min. The patient is currently asymptomatic without palpitations. The patient is immediately connected to a cardiac monitor which shows normal sinus rhythm. Over the course of the night, the monitor demonstrates 3 more episodes of the rhythm shown below in Figure A, with associated palpitations and lightheadedness. Blood pressure and oxygen saturation are stable during these episodes. The patient is given intravenous magnesium and diltiazem after the decision is made to also start medical therapy.	Yes	pharmacotherapy	9	The patient is given intravenous magnesium and diltiazem after the decision is made to also start medical therapy.	The patient is given intravenous magnesium and amdiodarone after the decision is made to also start medical therapy.
ms-473	A 65-year-old man is hospitalized after undergoing operative fixation of a left distal radius fracture due to a fall. On postoperative day 1, he reports having several episodes of palpitations with associated lightheadedness. He denies any chest pain and states that these episodes last for a few seconds each before resolving. On exam, his temperature is 98.4 F (36.9 C), blood pressure is 124/76 mmHg, pulse is 94/min, and respirations are 12/min. The patient is currently asymptomatic without palpitations. The patient is immediately	No	NA	NA	NA	NA

	connected to a cardiac monitor which shows normal sinus rhythm. Over the course of the night, the monitor demonstrates 3 more episodes of the rhythm shown below in Figure A, with associated palpitations and lightheadedness. Blood pressure and oxygen saturation are stable during these episodes. The patient is given intravenous magnesium. The decision is made to also start medical therapy. Amiodarone is started.					
ms-503	A 14-year-old female notes that while her fever, malaise, and a sore throat have dissipated a week ago, her hacking cough persists. A chest radiograph (Figure A) demonstrates a streaky infiltrate in her right lung that appears much worse than the symptoms she is experiencing. Patient is given vancomycin after a cold agglutination test conducted on her blood shows clumping together when placed at 4 degrees Celsius.	Yes	Treatment	2	Patient is given vancomycin after a cold agglutination test conducted on her blood shows clumping together when placed at 4 degrees Celsius.	Patient is given azithromycin after a cold agglutination test conducted on her blood shows clumping together when placed at 4 degrees Celsius.
ms-504	A 14-year-old female notes that while her fever, malaise, and a sore throat have dissipated a week ago, her hacking cough persists. A chest radiograph (Figure A) demonstrates a streaky infiltrate in her right lung that appears much worse than the symptoms she is experiencing. A cold agglutination test conducted on her blood shows clumping together when placed at 4 degrees Celsius. Patient is given azithromycin.	No	NA	NA	NA	NA

Table S4 Cohen's Kappa Test for the Evaluation of Eight Large Language Models Based on the Evaluation Model of GPT 4

Models	Cohen’s Kappa value
GPT 4	0.939
GPT o1	0.942
DeepSeek V3	0.957
DeepSeek R1	0.958
DeepSeek-R1-Distill-Qwen-32B	0.962
DeepSeek-R1-Distill-Qwen-14B	0.960
Qwen-32B-Instruct	0.909
Qwen-14B-Instruct	0.936

Table S5 Number of errors in medical records detected by eight large language models

Error Types	Correct answer	GPT 4	GPT o1	Deepseek V3	Deepseek R1	Qwen32 distill	Qwen14 distill	Qwen32 instruct	Qwen14 instruct
Causal Organism	10	10	14	4	12	6	11	13	17
Diagnosis	115	168	142	110	160	94	114	190	163
Management	96	130	46	35	99	26	52	122	150
Pharmacotherapy	35	119	15	59	107	75	55	5	10
Treatment	50	29	100	78	23	25	45	141	78

Table S6 Number of erroneous and correct medical records detected by eight large language models

Error Types	Correct answer	GPT 4	GPT o1	Deepseek V3	Deepseek R1	Qwen32 distill	Qwen14 distill	Qwen32 instruct	Qwen14 instruct
Causal Organism	10	7	6	4	7	3	3	7	4
Diagnosis	115	96	99	77	104	55	57	95	78
Management	96	48	22	20	44	12	13	38	45
Pharmacotherapy	35	29	8	19	23	15	12	1	3
Treatment	50	10	32	21	7	8	15	32	25

Table S7 Model Precision, Recall, F1 Score, Accuracy for eight large language models in different error types

Error Types	GPT 4	GPT o1	Deepseek V3	Deepseek R1	Qwen32 distill	Qwen14 distill	Qwen32 instruct	Qwen14 instruct
Causal Organism								
Precision	0.700	0.600	0.400	0.700	0.300	0.300	0.700	0.400
Recall	0.700	0.429	1.000	0.583	0.500	0.273	0.538	0.235
F1 score	0.700	0.500	0.571	0.636	0.375	0.286	0.609	0.296
Diagnosis								
Precision	0.835	0.861	0.670	0.904	0.478	0.496	0.826	0.678
Recall	0.571	0.697	0.700	0.650	0.585	0.500	0.500	0.479
F1 score	0.678	0.770	0.684	0.756	0.526	0.498	0.623	0.561
Management								
Precision	0.500	0.229	0.208	0.458	0.125	0.135	0.396	0.469
Recall	0.369	0.478	0.571	0.444	0.462	0.250	0.311	0.300
F1 score	0.425	0.310	0.305	0.451	0.197	0.176	0.349	0.366
Pharmacotherapy								
Precision	0.829	0.229	0.543	0.657	0.429	0.343	0.029	0.086
Recall	0.244	0.533	0.322	0.215	0.200	0.218	0.200	0.300
F1 score	0.377	0.320	0.404	0.324	0.273	0.267	0.050	0.133
Treatment								
Precision	0.200	0.640	0.420	0.140	0.160	0.300	0.640	0.500
Recall	0.345	0.320	0.269	0.304	0.320	0.333	0.227	0.321
F1 score	0.253	0.427	0.328	0.192	0.213	0.316	0.335	0.391

All Error Types								
Precision	0.621	0.546	0.461	0.605	0.304	0.327	0.565	0.507
Recall	0.499	0.679	0.610	0.587	0.557	0.490	0.445	0.452
F1 score	0.553	0.605	0.525	0.596	0.393	0.392	0.498	0.478
Accuracy	0.477	0.629	0.566	0.572	0.511	0.472	0.405	0.422

Table S8 Variance inflation factors for assembled large language models

Error Types	Models	VIF
Causal Organism	GPT 4	1.039
	GPT o1	1.457
	Deepseek R1	1.379
	Qwen32instruct	1.065
Diagnosis	GPT 4	1.847
	GPT o1	2.101
	Deepseek V3	1.627
	Deepseek R1	1.567
	Qwen32instruct	1.479
	Qwen14instruct	1.394
Management	GPT 4	1.000
Pharmacotherapy	GPT 4	1.089
	Deepseek R1	1.089
Treatment	GPT 4	1.359
	Qwen32instruct	1.359
All error types	GPT 4	1.001
	Deepseek R1	1.001

Table S9 Hyper-parameter tuning for logistic regression models

Error Types	Hyper-parameter tuning
Causal Organism	C: 1,
	penalty: 'l2',
	solver: 'saga'
	max_iter=1000,
	random_state=666,
	scoring='f1',
	cv=5,
	n_jobs=-1
Diagnosis	C: 10,
	penalty: 'l2',
	solver: 'liblinear'

	max_iter=1000,<br>random_state=666,<br>scoring='f1',<br>cv=5,<br>n_jobs=-1
Management	C: 0.1,<br>penalty: 'l2',<br>solver: 'liblinear'<br>max_iter=1000,<br>random_state=666,<br>scoring='f1',<br>cv=5,<br>n_jobs=-1
Pharmacotherapy	C: 1,<br>penalty: 'l1',<br>solver: 'liblinear'<br>max_iter=1000,<br>random_state=666,<br>scoring='f1',<br>cv=5,<br>n_jobs=-1
Treatment	C: 0.001,<br>penalty: 'l2',<br>solver: 'liblinear'<br>max_iter=1000,<br>random_state=666,<br>scoring='f1',<br>cv=5,<br>n_jobs=-1
All error types	C: 10,<br>penalty: 'l2',<br>solver: 'saga'<br>max_iter=1000,<br>random_state=666,<br>scoring='f1',<br>cv=5,<br>n_jobs=-1
